# Post-stapedotomy granulomatous reaction

**DOI:** 10.1590/S1808-86942010000100024

**Published:** 2015-10-17

**Authors:** Fernando Kaoru Yonamine, Danilo Kanashiro Segalla, Marcos Luiz Antunes

**Affiliations:** 1 Medical resident in Otorhinolaryngology, Otorhinolaryngology and Head & Neck Surgery Department, Sao Paulo Federal University, Paulista Medical School (EPM); 2 Medical resident; 3 Doctorate in Otorhinolaryngology, Sao Paulo Federal University, EPM. Coordinator of the Otorrinolaringology Unit of the Diadema State Hospital. Diadema State Hospital, Otorhinolaryngology and Head & Neck Surgery Department, Sao Paulo Federal University, EPM

**Keywords:** stapedectomy, granuloma, vertigo

## INTRODUCTION

Since the advent of stapedotomy and its use in the treatment of otosclerosis, a few complications have been reported in the literature. Among them, the granulomatous reaction, described by Harris and Weiss in 1962,^1^ is rare. It is an excessive inflammation that forms granulation tissue around the prosthesis and the oval windows.^2^

Although the etiology is uncertain, several authors believe that the main cause is a foreign body reaction to the material used in filling the oval windows.^3^

The post-stapedotomy incidence is 0.07% and the post-stapedectomy incidence is 0.1%; it generally manifests after surgery as sensorineural dysacusis and vertigo. It may be confirmed by exploratory tympanotomy to visualize granulation tissue around the prosthesis and the oval window in a symptomatic patient.^3^

## CASE REPORT

**Figure 1:**
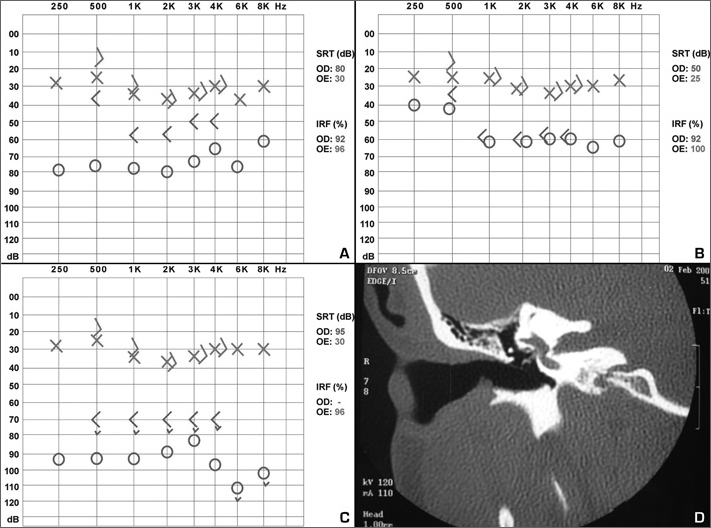
A: Preoperative audiometry. B: Audiometry two weeks after stapedotomy. C: Audiometry one month after stapedotomy. D: CT of the right temporal bone, coronal section, showing soft tissue density content in the oval window and around the prosthesis.

A white female patient aged 35 years complained of progressive bilateral hypoacusis during the last seven years, worse to the right. There was no significant history of disease. Her mother had bilateral hearing loss of unknown cause. There were no findings on the physical and otorhinolaryngological examination. Audiometry showed mild mixed hearing loss with a 5–10 dB gap at low frequencies and 30 dB SRT to the left, and moderate to severe mixed loss with a 20–35 dB gap and 80 dB SRT to the right (Fig. 1A); the speech recognition index and tympanometry were within normal limits, and the stapedian reflexes were absent. The patient underwent stapedotomy and placement of a Teflon prosthesis in the right ear; the oval window was filled with 1 ml of blood; no complications ensued. Post-operatively, the patient presented intense vertigo and the hearing loss remained. Vertigo diminished partially and hearing improved after treatment with an antivertiginous drug and corticosteroids (Fig. 1B). Two months of medication resulted only in partially regression of vertigo; but hearing worsened significantly. Computed tomography of the temporal bones revealed apparently well located soft tissue density material in the oval window and around the prosthesis (Fig. 1D). A revision of the stapedotomy was carried out, which showed granulation tissue in the oval window. The prosthesis was removed with the granuloma. One month later the patient had improved partially from the vertigo, but hearing was worse. Audiometry showed profound sensorineural hearing loss to the right, with 95 dB SRT (Fig. 1C).

## DISCUSSION

The disease should be suspected in patients presenting postoperative vertigo and persistent sensorineural dy-sacusia within six months of surgery.^2^ The differential diagnosis is made with perilymphatic fistula and stapedotomy long prosthesis. High resolution computed tomography may be used, since this method makes it possible to identify these conditions.^4^

A foreign body reaction has been suggested as the hypothesis, although autoimmune reactions, infection and local inflammation have not been discarded.^1^ The most common filling materials in stapedotomy that have been associated with granuloma are blood and gelfoam; in stapedectomy, these materials are fat and gelfoam.^3^ They are used to fill in the oval window and to decrease the risk of a perilymphatic fistula. An animal experimental model showed that gelfoam placed in the open oval window niche caused injury to the basilar membrane on histology.^5^

Surgical treatment may be exploratory tympanotomy with removal of the granuloma and the prosthesis; a second prosthesis may be placed, or the granuloma may be removed only. Clinical therapy is done with corticosteroids and antibiotics.^3^

Even though there are no published papers specifically on the treatment of granulomas, several authors, based on their experience, have obtained better results by combining corticosteroids and early revision with removal of the granuloma and placement of a new prosthesis.^3^,^6^

## FINAL COMMENTS

Granulomatous reaction following stapedotomy/stapedectomy is rare, rather annoying for patients, and frustrating for surgeons. Unfortunately, there are not predictive patterns for its occurrence. We should be ready to deal with this situation, notwithstanding its rarity.
